# Editorial

**Published:** 1849-10

**Authors:** 


### TO OUR SUBSCRIBERS.

Those who are in arrears, will have an opportunity, on the receipt of the present number, to remit for two years. We hope this opportunity will be generally complied with, as it is entirely out of the question to send around an agent to make collections, and such a work cannot be sustained without prompt payment.—[J. T. Cin. Ed.

